# Treatment of patients with refractory metastatic cancer according to molecular profiling on tumor tissue in the clinical routine: an interim-analysis of the ONCO-T-PROFILE project

**DOI:** 10.18632/genesandcancer.121

**Published:** 2016-09

**Authors:** Andreas Seeber, Guenther Gastl, Christian Ensinger, Gilbert Spizzo, Wolfgang Willenbacher, Florian Kocher, Christoph Leitner, Ella Willenbacher, Arno Amann, Normann Steiner, Wolfgang Eisterer, Andreas Voss, Kenneth Russell, Heinz Zwierzina

**Affiliations:** ^1^ Department of Haematoloy and Oncology, Innsbruck Medical University, Austria; ^2^ Laboratory for Experimental Oncogenomics, Tyrolean Cancer Research Institute, Austria; ^3^ Department of Pathology, Innsbruck Medical University, Austria; ^4^ Haematooncological Day Hospital, Hospital of Merano, Italy; ^5^ Caris Life Sciences, Basel, Switzerland

**Keywords:** molecular profiling, cancer, personalized medicine, caris life sciences, next generation sequencing

## Abstract

**Introduction:**

Patients with refractory metastatic cancer have been shown to benefit from molecular profiling of tumor tissue. The ONCO-T-PROFILE project was launched in March 2014 at the Innsbruck Medical University. Within 2 years our project aims to recruit 110 patients with stage IV cancer refractory to standard therapy. Our data presented here are based on an interim-analysis.

**Methods:**

Tumor tissue specimens were submitted for molecular profiling to the certified laboratory (Caris Life Science, USA). Druggable tumor targets were selected based on biomarker status to agents with potential clinical benefit. Clinical benefit was defined as a PFS ratio (=PFS upon treatment according to the molecular profile/ PFS upon the last prior therapy) ≥ 1.3.

**Results:**

As of April 2015, tumors from 50 patients have been molecularly profiled and one or more targets were detectable in 48 specimens (98%). So far, 19 (38%) patients have been treated according to their molecular tumor profile. To date, 8 (42%) patients have reached a PFS ratio of ≥ 1.3.

**Conclusions:**

We could show that molecular profiling is feasible in the clinical routine. A proportion of patients might benefit from an individualized treatment approach based on molecular profiling. As a result, we will proceed to enroll patients in ONCO-T-PROFILE.

## INTRODUCTION

The standard treatment for metastatic cancer patients is based on the histopathology of tumor tissues. However, in the last years a new wave of knowledge about the genomic and molecular structure of cancer cells has entered routine clinical practice. This has led to the approval of many drugs capable to target specific molecular alterations in a certain tumor, e.g., gefitinib in EGFR-mutated non-small cell lung cancer [1] or trastuzumab in HER2-amplified or overexpressed breast cancer [2]. The Cancer Genome Atlas Network revealed that specific genomic mutations and alterations are expressed not only in one certain histopathological defined tumor (i.e. BRAF in melanoma) but also in tumors deriving from other origins [3, 4]. This led to the consideration to abandon “classic” organ-specific histopathological analyses and to diagnose and treat patients according to their molecular profile [5].

In 2006, a feasibility analysis showed that by using immunohistochemistry (IHC) and oligonucleotide microarrays, a druggable target could be found in 98% of profiled cases. Therefore, a pilot-study was conducted to analyze the effect of targeted therapy according to the molecular profile of a metastatic tumor. In total, 86 patients with different refractory metastatic cancers showed a response rate of 27% with a 30% prolonged PFS (ePFS > 1.3) compared with the PFS after previous treatment [6]. A further study investigated the survival benefit of molecular characterization in 25 breast cancer patients with more than 3 prior treatment lines. In total, 44% (*n* = 11) showed a PFS ratio of > 1.3 [7].

The first randomized trial to investigate the value of treatment according to molecular profiling was the SHIVA trial. This phase II trial enrolled 195 patients with any kind of metastatic tumors refractory to standard treatments and randomly assigned to treatment according to molecular profiling or physicians’ choice. Surprisingly, no advantage in terms of survival could be shown for patients treated with regimens based on molecular phenotyping [8]. The majority of treatment associations (74%) in this study was not based on clinical data but followed hypotheses based on preclinical data.

In the last few years so-called “basket” trials were designed to target patients with a specific genomic alteration independent of the histology-based diagnosis. A phase II trial investigated the effect of vemurafenib in BRAF-mutated non-melanoma tumors. The response rate was 42% and the median PFS was calculated at 7.3 months. Interestingly, the activity was stronger in some entities, such as non-small cell lung cancer, but lower in others, such as ovarian or colorectal cancer [9]. It was shown later, that in colorectal cancer combination therapies of vemurafenib or dabrafenib with an EGFR directed monoclonal antibody [10] or with a MEK inhibitor [11] could successfully be used to treat patients with a BRAF mutation. These data show that the effect of molecularly-based treatment allocation needs further refinement. For this reason we established the “ONCO- T-Profile” project. The aim of this project is to treat 110 patients with different refractory tumors according to their molecular profile analyzed by methods such as next- generation sequencing (NGS) or immunohistochemistry (IHC). Here, we present the data of the interim analysis.

## PATIENTS AND METHODS

### The ONCO-T-PROFILE project

ONCO-T-PROFILE was initiated in March 2014 at the Department of Haematology and Oncology of the Innsbruck Medical University. The aim is to treat 110 patients with advanced solid tumors with no further standard antineoplastic treatment options available, in a personalized manner. Therefore, after obtaining informed consent, a mandatory biopsy or an archieved sample from the resection of the tumor is collected and sent to a certified laboratory (Caris Life Sciences, Phoenix, AZ, USA) where multi-modal molecular profiling is performed. After approximately two weeks, a detailed case report with illustration of mutations and potential targetable structures is sent back to the investigator site in Innsbruck, Austria. The results of this molecular profiling are discussed among the treating physicians, Caris Life Sciences and an expert panel of the ONCO-T-PROFILE team. According to blood tests and performance status of the patient, a personalized therapy approach may be recommended by the treating physician. Two to three cycles or 2-3 months of therapy should be given before a restaging by imaging is performed.

The objective of this project is to compare the progression-free survival (PFS) obtained by the experimental therapy with the PFS of the last treatment on which the same patient experienced a progress. As such, each patient is her/his own historical control.

### Patient`s selection

Patients older than 18 years with a histologically confirmed metastatic and recurrent solid tumor that failed standard treatment are eligible for this project. Formalin- fixed paraffin-embedded (FFPE) tumor material to perform molecular profiling must be available. Patients with an Eastern Cooperative Oncology Group (ECOG) Performance status between 0 and 2 are allowed to participate. Furthermore, a life expectancy of more than 3 months, adequate liver, renal and bone marrow functions, and a written informed consent are required.

### Molecular profiling

Molecular Profiling (MP) is performed on FFPE specimens using the “Caris Molecular Intelligence” (CMI) service. For that, multiple different standard platforms and methods, including next-generation sequencing (NGS), immunohistochemistry (IHC) and in-situ hybridizations (FISH/CISH), are used. The type of analyses performed and the specific biomarkers tested depended on the amount of tissue sample available.

IHC analysis was performed on formalin-fixed paraffin-embedded (FFPE) tumor samples using commercially available certified detection kits, automated staining techniques including BenchMark XT (Ventana Medical Systems, Inc., Tucson, AZ) and Autostainer Link 48 (Dako North America, Inc., Carpinteria, CA), and commercially available antibodies.

FISH and CISH was used to evaluate HER2/neu [HER2/CEP17 probe], EGFR [EGFR/CEP7 probe], and cMET [cMET/CEP7 probe] (Vysis PathVysion FISH assay, Abbott Laboratories, Abbott Park, IL). HER2/ neu and cMET status were evaluated by CISH using the INFORM HER2 Dual ISH DNA Probe Cocktail, and the Chromosome 7 DIG Probe (Ventana Medical Systems, Inc., Tucson, AZ). The same scoring system was applied as for FISH. Either the absolute gene copy number in tumor cells or a gene: CEP17 signal ratio was used to score results in both methods.

HER2 CISH test was carried out using the INFORM DUAL HER2 ISH Assay (Ventana). Control was CEP17. Cutoff was HER2/CEP17 ratio > = 2.0. cMET CISH was carried out using a probe specific for cMET pericentromeric region of chromosome 7 (Ventana). Positivity for increased gene copy number for cMET CISH has been defined as mean of ≥5 copies of MET gene per cell in NSCLC, because the gene copy number threshold for other tumor types has not been determined. TOP2A CISH was carried out using a probe specific for TOP2A pericentromeric region of chromosome 17 (Ventana). Control was CEP17. Cutoff was TOP2A/CEP17 ratio >= 2.0 or the presence of the mean of ≥ 6 copies of the TOP2A in cancer cells. EGFR CISH was carried out using a probe specific for EGFR - pericentromeric region of chromosome 7 (Ventana).

Direct NGS analysis was performed on genomic DNA isolated from FFPE tumor samples using the MiSeq platform (Illumina, Inc., San Diego, CA). Specific regions of 45 genes of the genome were amplified using the TruSeq Amplicon Cancer Panel (Illumina, Inc., San Diego, CA). Mutation analysis by Sanger sequencing included selected regions of BRAF, KRAS, cKIT, EGFR, and PIK3CA genes and was performed by using M13- linked PCR primers designed to amplify target sequences. The depth of coverage was >1000X. Depth of coverage in DNA sequencing refers to the number of times a nucleotide is read/analyzed during the sequencing process. Coverage is the average number of reads representing a given nucleotide in the reconstructed sequence. 100% of NGS samples were microdissected after pathologist identification of tumor cells.

### Statistics

According to the study of Von Hoff *and colleagues* [6] a PFS ratio of ≥ 1.3 is warranted to display a clinically relevant benefit of experimental therapy. Progression free survival ( = PFS) was defined as time of treatment start to date of tumor progression. The PFS ratio was defined as PFS under molecular guided therapy / previous PFS on which patient progressed. To allow for benchmarking our results we decided on using the same threshold value defining a positive outcome.

## RESULTS

### Patient population

**Figure 1:**
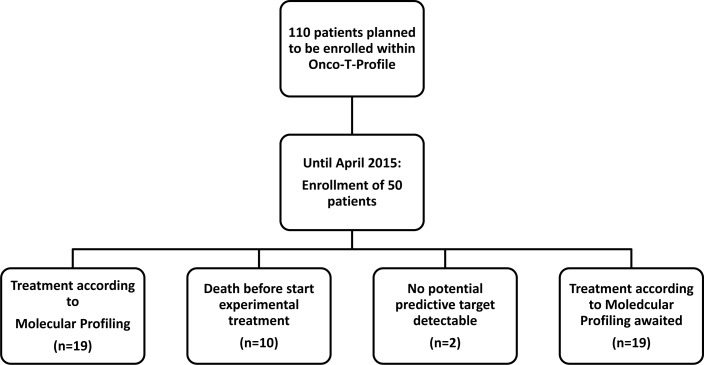
Consort diagram of the interim analysis of the ONCO-T-PROFILE project

From March 2014 until April 2015, 50 patients with refractory solid cancer were enrolled in our ONCO-T- PROFILE project. As illustrated in Figure 1, so far, 19 patients were treated according to molecular profiling. Only in 2 of 50 patients (4%) we were not able to detect any potential targetable alteration. Twenty-nine patients (58%) are currently on standard therapy with already performed tumor profiling that will allow a potential switch to experimental treatment if their performance status and blood tests allow. Of the 50 patients enrolled within the ONCO-T-PROFILE program so far, breast cancer (*n* = 8, 16%) was the most dominant tumor type, followed by colorectal and ovarian cancer (both *n* = 7, 14%). Five patients (10%) suffered from lung cancer (non- small cell lung cancer: *n* = 4, 8%; small cell lung cancer: *n* = 1, 2%). The other patients suffered from pancreatic cancer (*n* = 3; 6%), sarcoma (*n* = 3; 6%), neuroendocrine tumor/carcinoma (*n* = 3; 6%), gastric cancer (*n* = 2; 4%) and cholangiocellular-carcinoma (*n* = 2, 4%). The rest of the patients had other tumors, such as adrenocortical or endometrial carcinoma (in total 10 patients, 20%; see table 1). The mean age of treated patients was 57 years (range: 21-83 years) and the majority was female (*n* = 32, 64%). Seventeen patients (34%) had a good ECOG PS of 0, 21 patients (42%) had an ECOG PS of 1 and 12 patients (24%) had an ECOG PS of 2.

**Table 1:** Clinical characteristics of the first 50 patients enrolled in the ONCO-T-PROFILE project

Baseline Characteristics	No. of Patients	Percentage
Total patients	50	100
**Age, years**		
Mean	57	
Range	21-83	
**Sex**		
Female	32	64
Male	18	36
**ECOG PS**		
0	17	34
1	21	42
2	12	24
**Tumor entity**		
**GI Cancer**	14	28
CRC	7	
Pancreatic	3	
Gastric	2	
CCC	2	
**Gynaecologic Cancer**	20	40
Breast Cancer	8	
Ovarian Cancer	7	
Others	5	
**Sarcoma**	3	6
**NET/NEC**	3	6
**Lung Cancer**	5	10
NSCLC	4	
SCLC	1	
**Other Malignancies**	5	10

### The effect of molecularly based treatment

In Table 2 the clinical data of the 19 patients treated with experimental regimens according to the molecular profiling results are listed. Mean age was 76 years (range: 25-83 years) and patients had received between 1 and 8 treatment lines prior to molecular guided therapy. Eight patients had an ECOG PS of 0, 6 patients an ECOG PS of 1, and further 5 patients an ECOG PS of 2.

**Table 2:** Clinical characteristics and administered therapies according to the molecular profiling of the first 19 patients

Patient ID	Age [years]	ECOG PS	Cancer Type	No. of cycles before ONCO-T-PROFILE	Experimental Regimen	PFS *vs*. ePFS [days]	PFS Ratio > 1.3
P1	44	2	CRC	8	nab-Paclitaxel + Gemcitabine*	86 *vs*. 56	No
P2	65	0	CRC	7	Doxorubicin*	98 *vs*. 62	No
P4	68	0	Sarcoma, NOS	4	Paclitaxel + Gemcitabine	44 *vs*. 237	Yes
P3	47	2	Adrenocortical Carcinoma	5	nab-Paclitaxel*	56 *vs*. 30	No
P15	37	1	Breast Cancer	8	Exemestane + Everolimus	184 *vs*. 249	Yes
P5	67	1	Liposarcoma	5	Gemcitabine	269 *vs*. 93	No
P18	64	0	Breast Cancer	5	Carboplatin + Gemcitabine	220 *vs*. 250	No
P7	46	2	Pancreatic Cancer	2	Regorafenib*	36 *vs*. 56	No
P8	55	2	SCLC	5	Irinotecan*	54 *vs*. 68	No
P9	67	1	NET	2	Topotecan	89 *vs*. 194	Yes
P11	69	0	NSCLC	4	Gemcitabine	62 *vs*. 135	Yes
P6	83	0	Endometrial Carcinoma	2	Liposomal Doxorubicin	243 *vs*. 74	No
P12	49	1	Gastric Cancer	3	Epirubicin + Docetaxel	204 *vs*. 100	No
P10	51	0	Breast Cancer	2	Exemestane + Everolimus	17 *vs*. 124	Yes
P16	64	1	Endometrial Carcinoma	2	Liposomal Doxorubicin	71 *vs*. 156	Yes
P14	45	0	Breast Cancer	3	Exemestane + Everolimus	242 *vs*. 325	Yes
P17	25	0	Ovarian Cancer	1	Everolimus	83 *vs*. 156	Yes
P19	67	2	CCC	5	nab-Paclitaxel*	264 *vs*. 57	No
P13	57	1	CRC	3	Regorafenib	223 *vs*. 176	No

Abbreviations: CRC: colorectal cancer, NOS: not otherwise specified, SCLC: small cell lung cancer, NSCLC: non-small cell lung cancer, NET: neuroendocrine tumor, CCC: cholangiocellular-carcinoma.

* therapies which are not used in the respective indication

**Figure 2:**
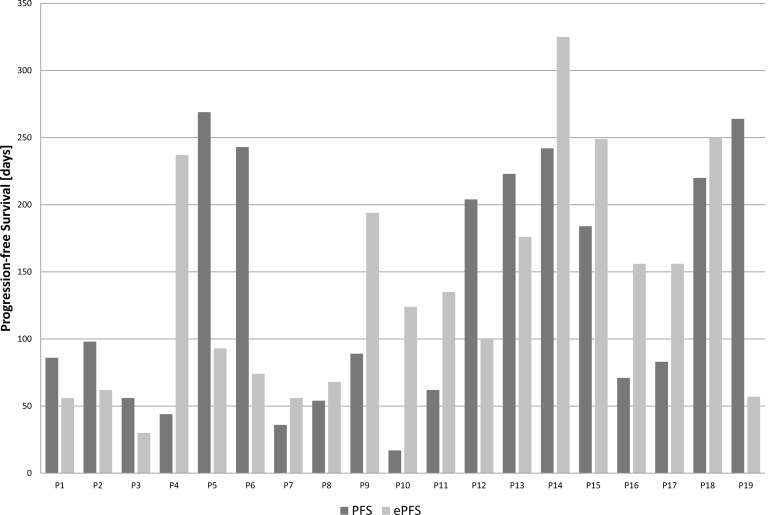
Progression-free survival comparison between the experimental treatment (ePFS; grey) according to molecular profiling and the prior therapy (PFS; black) of the first 19 patients treated within the ONCO-T-PROFILE project

In Table 2 and Figure 2 the effect of the experimental treatment according to the molecular characterization of the tumor samples is displayed. Of the first 19 patients treated within ONCO-T-PROFILE, 8 patients (42%) had a PFS ratio > 1.3 (range: 0,22 to 7,29; median: 1,26).

### Genomic alterations detected by next-generations sequencing

**Figure 3:**
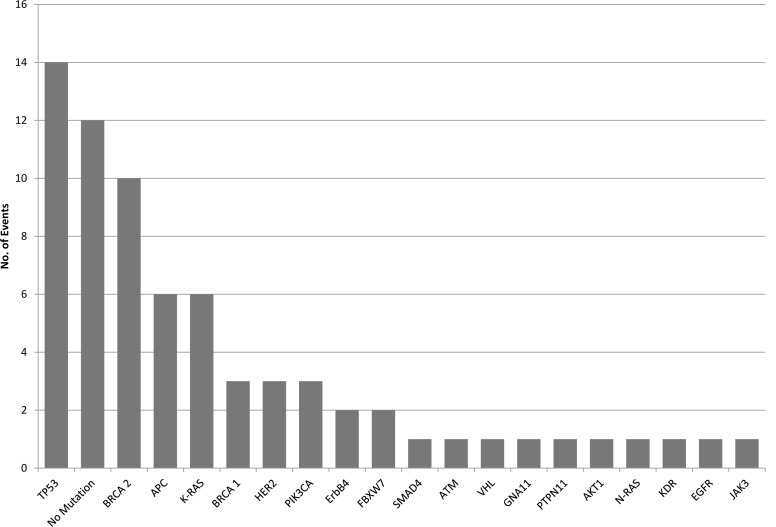
Mutational aberrations detected by next-generation sequencing of the 50 patients within the ONCO-T-PROFILE project

In the 50 patients analyzed by next-generation sequencing (NGS) 59 mutations were detected (Figure 3). In 12 patients (24%), no relevant pathological alteration could be found by NGS. The most common detectable gene mutation was located in TP53 (*n* = 14, 28%) followed by BRCA 2 mutation (*n* = 10, 20%). APC and K-RAS mutations, typically colorectal cancer-associated, were seen in 6 cases (12%), respectively. In two patients (4%) BRCA 1, PIK3CA and HER2 alterations, which are commonly found in breast cancer, were observed. In 4% (*n* = 2) of patients ErbB4 and FBXW7 mutations were detected. Rare mutations such as AKT1 or JAK3 were seen in one patient (2%), respectively.

## DISCUSSION

To the best of our knowledge, ONCO-T-PROFILE is the first ongoing project to investigate molecular-based targeted therapy in patients with refractory solid tumors in the clinical routine. In this interim-analysis, we describe the first 50 molecularly typed patients enrolled at the Innsbruck Medical University of whom 19 patients were treated according to their molecular profile. In 48 of 50 patients at least one potential target could be found. Up to April 2015 we have treated 19 of 50 patients using drugs which were predicted to be of potential benefit to the patient. The other 29 patients are still on standard treatment and planned to receive therapy according to molecular profiling at the time of progression, presupposed performance status will allow. This personalized approach resulted in 42% of patients achieving a 30%-prolonged progression-free survival (PFS) compared to the previous PFS of the last standard therapy. In these patients, who were treated with drugs that would not otherwise have been chosen, even prolonged responses could be observed. This is in line with the results obtained by the group of Von Hoff D *et al* which performed a study investigating the clinical effect of personalized therapy using IHC, FISH and oligonucleotide microarray in a heavily pretreated mixed patient cohort. They found a prolonged PFS ratio in 27% of patients who received experimental treatment. Although our study used more modern and more sensitive methods (such as next-generation sequencing) compared to Von Hoff D *et al*, we could not find a higher rate of potentially druggable targets (96% *vs.* 98%) [6].

In recent years, personalized therapy has got into the focus of clinical research since different collaborative groups such as the Cancer Genome Atlas Network have not only found mutations but also overexpression of glycoproteins on the surface of tumor cells [12-14]. This led to the development of novel agents targeting specific structures on the tumor membrane, such as EGFR or HER2 [1;2]. In our cohort we used next-generation sequencing (NGS) to detect the most common mutated genes. The majority of patients enrolled had breast and colorectal cancer, so that TP53 mutation was the most common observed mutation followed by BRCA-2, K-RAS and APC.

In this interims analysis we wanted to analyze whether such a profiling was feasible in the clinical routine or not. We could demonstrate that molecular profiling in patients with an ECOG PS 0-2 is practicable and might result in a substantial clinical benefit. Ten patients (20%) were already lost prior to experimental treatment due to worsening condition. Notably, 7 of those 10 patients were in an ECOG PS 2 and other 3 patients in an ECOG PS 1. The results so far are not matured enough, but we hypothesize that mainly patients in a good performance status are able to gain a benefit of such molecularly based treatments.

The impact of molecular profiling in metastatic tumor patients remains controversial. The only randomized phase II trial evaluating treatment according to molecular profiling compared to physicians` choice failed to show prolonged survival in patients treated according to molecular phenotyping [8]. However, we and others provide first data reflecting the potential benefit of a personalized approach in selected patients [6;7]. These controversial data might be explained due to a different approach to molecular profiling. In the SHIVA trial analysis was mainly performed by next- generation sequencing, gene copy number alterations and immunohistochemistry was used for hormone receptor status and to confirm any deletions or amplifications. In the certified laboratory of Caris Life Sciences, where the molecular profiling of our and other studies [6;7] was performed, In the molecular profiling used for ONCO-T- PROFILE a wider panel of potential predictive markers was used. In addition, the predictive treatment associations in CMI are based on published clinical literature and are regularly updated to provide the most recent information. The treatment algorithms used in the SHIVA study were fixed and the majority was based on preclinical data or mechanistic associations. As such, we and others had more proteins available to target with specific treatment. Moreover, the end point of the SHIVA study was progression-free survival (PFS) and not the ratio between experimental PFS and the PFS on the last therapy line, as we used here. These data are missing from SHIVA but will be presented soon [15].

In conclusion, this interim-analysis of the ONCO- T-PROFILE project shows the feasibility of molecular profiling in patients with advanced solid tumors refractory to standard treatments in the daily routine. A subset of patients whom underwent experimental therapy showed a prolonged PFS compared to the PFS of their previous treatment line, confirming a potential benefit of personalized targeted therapy. A complete analysis of the 110 patients within the ONCO-T-PROFILE is expected in 2017. Furthermore, due to the feasibility of molecular profiling we will broaden our spectrum of eligible malignancies comprising haematologic diseases such as multiple myeloma.
